# Art and Esthetics as Applied to Prosthetics*Read before the annual meeting of the Central Dental Association of Northern New Jersey, Newark, March 1, 2 and 3, 1921. Reprinted from *Cosmos*, December, 1921.

**Published:** 1922-01

**Authors:** P. C. Lowery

**Affiliations:** Detroit


### ART AND ESTHETICS AS APPLIED TO PROSTHETICS*

BY P. C. LOWERY, D.D.S., DETROIT

In order to establish the proper hypothesis for this article, I shall take the liberty of refreshing your memory with the definitions of esthetics and art. Dental esthetics, the science which deduces from nature the rules and principles of facial and dental art; dental art, the theory or practice of esthetics in the expression of beauty in form, size, arrangement and hue of the teeth and facial expression.

The term "esthetics and art" generally is thought to be applied to lend dignity to a particular phase of dentistry. It is obvious that its real value is not in theory, but in practice.

In considering this science, the prevailing tendency has been to create an artificial standard. In this, I believe, we have been blinded to the fact that the most

* Read before the annual meeting of the Central Dental Association of Northern New Jersey, Newark, March 1, 2 and 3, 1921. Reprinted from *Cosmos*, December, 1921.

authentic guide is a careful observance of the types which the majority of us meet in our every-day contact with the human family.

That the environment of the worker has its effect upon all art work is a well-known fact. Genius has the power to create its own illusion of environment, regardless of surroundings, hunger, cold, isolation, etc., but to those lacking this element, it is of the greatest importance to have a congenial atmosphere. As proof of this statement, one needs only to visit the homes of talented writers and painters in order to arrive at the conclusion that the pleasant surroundings were conducive to their best work.

Let us make a practical application of the principle. If we are to accomplish our best artistic results we must have congenial assistants. The office furnishings must be neat and attractive, not necessarily elaborate. We should develop in our patients an appreciation of the artistic value of the work.

Art is real and is governed by very tangible laws. It is safe to assume that knowledge and application of these laws are pertinent in establishing harmonious balance between tooth form, face form and arch form.

Dr. George Wood Clapp says, in effect: "Good practice in art demands that in any one composition there can be only one dominant theme. In our application of this law, the outline of the face seen full front in repose is the major premise of the composition and needs the upper central incisor of the same outline as the theme, to complete the cosmic whole. There must, however, be sufficient diversity of outline of teeth to avoid monotony."

The correct time and method to determine the indicated mold of tooth to be used in a prosthetic restoration is at the time that the mandible has been brought to its position of rest or relaxation. The occlusion rims should be in position, so that you have the desired facial expression and profile. The face should be seen full front view, and have glabella-nasion vertically over symphysismenti.

Direct your vision so that the level of your eye falls in a plane with the posterior border of the zygoma.

I believe that fewer forms and hues of teeth would meet our requirements and ultimately be the means of securing better esthetic results. From a chromatic stand point, there also should be a greater variance in the degree of saturation, ranging posteriorly from the centrals, the variation being one of intensity of a hue rather than a contrast of color. A hue guide having this gradation of hue in a sufficient number of anterior teeth to give adequate perspective would be extremely valuable.

In arranging teeth from an esthetic standpoint, the two factors to be given special consideration are the primary alinement and irregularities. All cases may be divided into three typal forms, which are subject to modification, and are typical of the square, tapering and ovoid primary alinements. I refer you to my article in the July issue of the *National Dental Journal,* 1920, pages 611 to 618, covering this subject. With this fundamental knowledge you should be able to successfully blend the modified cases.

The proper development of the bony framework of the face is dependent upon the exercise of force in thorough mastication of hard food.

The outline form of the arch, alinement of the teeth, the breadth of the condyle, and, in turn, the type of the face form are due to the degree of the development resulting from the exercise of this masticatory force. In edentulous cases the condyle or breadth in the region of the zygoma is the one constant we have from which to begin reconstruction.

With this knowledge before us, the following guide is offered as to whether there should be any irregularity of the anterior teeth. If the face is broad in the region of the zygoma in comparison to length, it indicates that there has been a full development of the dental arch and little if any irregularity, such as lapping or rotating of centrals or laterals. If the face is very broad in comparison to length, spacing of the anterior teeth is indicated, so as not to have the centrals too broad for the rest of the face and still have the distal surface of the cuspids in good position to the corners of the mouth. If the face is narrow and lacks full development, rotating or lapping of either or both centrals and laterals is indicated.

I recommend only the slightest irregularity in alinement, however, its degree or extent must depend upon the peculiarities of the case and the operator's artistic judgment. A knowledge of false perceptions or optical illusions is necessary to assure the retention of finest harmony between face form and tooth form in arranging the teeth. We must remember that it is not so much what we have, but what we do with what we have, that gives our work individuality.

Adherence to the principle commonly expressed as "the ideal arrangement of teeth," invariably leads to a stereotyped procedure. I believe that the law of harmony governs the arrangement of teeth for the individual case.

Some patients are reluctant to become edentulous and request the immediate insertion of dentures, for reasons of their pride in personal appearance. Aside from keeping our patients dentally presentable, this procedure has other merit, as it greatly reduces the mechanical irritant effect of dentures.

Knowing that the features of the mouth are easily distorted and difficult to restore, I suggest that dentures or temporary base plates be inserted in the mouth immediately after extraction, in order to maintain the facial muscle tone, and prevent collapse of the temporo-mandibular joint.

### ALVEOLECTOMY

In this procedure we must resort to alveolectomy. If done by one not familiar with this phase of oral surgery, the surgical interference or preparation of the mouth for dentures, instead of increasing the retentive power, may lessen it to a tremendous degree.

The subject of surgical preparation of the mouth has received considerable attention from the profession during the past year. When properly done it is conducive to success with dentures.

A commendable technic, for early insertion of dentures, is either the early method of impression taking before extraction, or Dr. Gillis' technic, described in the *Dental Summary* for September, 1920.

I prefer a procedure which has been in use for years, *i. e.*, having the molars and second bicuspids extracted prior to the rest of the teeth and allowing the ridge to become in good condition. My reasons for this preference are that the removal of the bicuspids and molars usually is due to a pathological condition, and I feel that it is better to remove only one or two teeth first. Later when the patient has had sufficient rest period to recover from the reaction, the remaining bicuspids and molars are removed. By not inserting the denture at this time, better drainage is established; by leaving the anterior teeth *in situ* the natural position of the mandible is retained, and at the same time the condition of the edentulous area improves, leaving a more permanent base for the reception of a denture.

Take impressions and register central occlusion before extraction of anterior teeth, recover casts, trim off teeth, set up and vulcanize dentures. It may be said that the trimming of teeth is only guesswork, but it affords immediate denture service, and retaining of the temporo-mandibular joint. In all cases where I have adopted this method I have found that the effort on the part of the patient in learning to wear dentures has been minimized. The patient after leaving the case in for a few hours will feel more comfortable with it in than out, the denture taking the place of a dressing to a wound. A rebasing is necessary at a later date.

Occasionally it is necessary to convince a patient that his or her appearance has not been materially altered by the surgical operation and insertion of these dentures. For this reason, I would suggest that photographic records and study models of all cases be made and retained for reference. Dr. V. C. Smedley says:

"The photographs should consist of one full front in repose, one full front smiling, one profile and one close up of the teeth if present, also if possible securing from the patient a photograph taken before the natural teeth were lost.

Rather than incur the delay and inconvenience so often experienced when a patient is sent to a professional photographer, I would suggest that each practitioner, anticipating doing this work, equip his office with a camera, especially adapted to this class of work, and consider portrait-making as necessary a part of his general education as the use of the X-ray.

By proper arrangement of mirrors a full front and profile view may be taken by one exposure. For an accurate profile record a life-size photograph of the outline profile is excellent. A photograph of your patient's appearance between twenty and thirty years of age is another valuable record to have and preserve for future use in restoring facial dimensions.

While it is true that the phase of dentistry known as "esthetics and art" has made gigantic strides in the past few years, still it is but in its infancy, and in each man present there is the nucleus of an idea for the improvement of this branch, which it is his privilege and duty to perfect for the benefit of the profession.

